# Active and Low-Cost Hyperspectral Imaging for the Spectral Analysis of a Low-Light Environment

**DOI:** 10.3390/s23031437

**Published:** 2023-01-28

**Authors:** Yang Tang, Shuang Song, Shengxi Gui, Weilun Chao, Chinmin Cheng, Rongjun Qin

**Affiliations:** 1 Geospatial Data Analytics Laboratory, The Ohio State University, Columbus, OH 43210, USA; 2 Department of Civil, Environmental and Geodetic Engineering, The Ohio State University, Columbus, OH 43210, USA; 3 Department of Electrical and Computer Engineering, The Ohio State University, Columbus, OH 43210, USA; 4 Translational Data Analytics Institute, The Ohio State University, Columbus, OH 43210, USA

**Keywords:** active hyperspectral imaging, spectrum-based recognition, sensing

## Abstract

Hyperspectral imaging is capable of capturing information beyond conventional RGB cameras; therefore, several applications of this have been found, such as material identification and spectral analysis. However, similar to many camera systems, most of the existing hyperspectral cameras are still passive imaging systems. Such systems require an external light source to illuminate the objects, to capture the spectral intensity. As a result, the collected images highly depend on the environment lighting and the imaging system cannot function in a dark or low-light environment. This work develops a prototype system for active hyperspectral imaging, which actively emits diverse single-wavelength light rays at a specific frequency when imaging. This concept has several advantages: first, using the controlled lighting, the magnitude of the individual bands is more standardized to extract reflectance information; second, the system is capable of focusing on the desired spectral range by adjusting the number and type of LEDs; third, an active system could be mechanically easier to manufacture, since it does not require complex band filters as used in passive systems. Three lab experiments show that such a design is feasible and could yield informative hyperspectral images in low light or dark environments: (1) spectral analysis: this system’s hyperspectral images improve food ripening and stone type discernibility over RGB images; (2) interpretability: this system’s hyperspectral images improve machine learning accuracy. Therefore, it can potentially benefit the academic and industry segments, such as geochemistry, earth science, subsurface energy, and mining.

## 1. Introduction

### 1.1. Background

Hyperspectral remote sensing is uniquely positioned to acquire abundant spectral information beyond normal optical image sensors and has been recognized as an important avenue to address challenges for many applications [1], such as environmental monitoring [2], mine exploration [3], precision agriculture [4], seed viability study [5], biotechnology [6], psychophysical studies [7], pharmaceuticals [8], and exploration of oil and gas [9]. In comparison to other techniques, for example, acoustic emission tomography [10,11,12] is an important monitoring method in the minefield utilizing a combination of active and passive sources. The hyperspectral camera does not require direct contact with the object’s surface; therefore, it can be applied to the broader field. For example, hyperspectral imaging has been used in agriculture to monitor the health of crops through remote sensing. In Australia, researchers use periodically scanned hyperspectral images to build early warning systems for disease epidemics by utilizing imaging spectrometers [13]. Compared to other broadband images (i.e., multispectral or normal RGB (red, green, blue) images), hyperspectral images can provide more detailed information about objects to facilitate more advanced applications. For example, they can be used to detect plants and their species [14,15], as well as extract detailed surface mining information [16,17,18].

**Figure 1:**
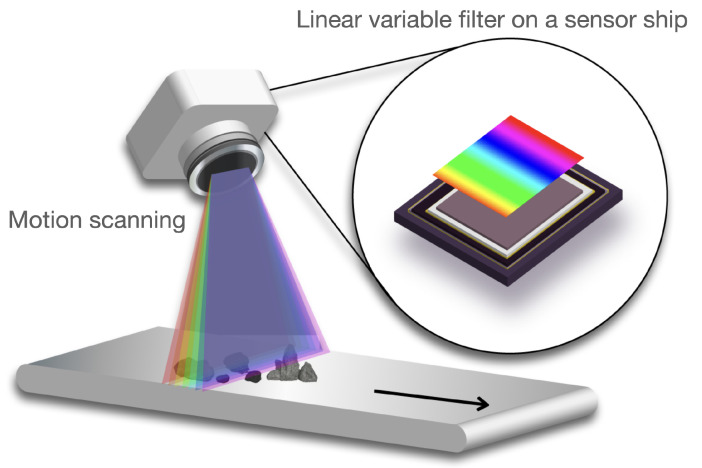
Linear variable filter motion scanning. An example of a linear variable filter on a sensor chip to achieve hyperspectral imaging. Imaging is achieved through motion-based scanning [23].

As with many other optical cameras, the majority of such hyperspectral imaging systems use a passive sensing scheme, which requires external lighting and operates primarily in daylight. It collects the reflected energies of natural light sources, such as solar radiation or ambient room lighting from the object. The sensor then splits the spectrums into individual bands; either through band filters [19] or optical splitters [20,21]. Due to the fact that hyperspectral cameras contain tens or hundreds of these bands, the mechanics of these filters/splitters are often difficult to implement, making these cameras more expensive and bigger than conventional cameras. To accommodate, many hyperspectral cameras adopt linear-array sensors through a linear-variable filter (concept figure shown in Figure 1) [22], in which different band filters are implemented at different lines of cells in the sensor chips to achieve hundreds of bands, whereas it requires the camera to collect images with a motion (i.e., linear scanning), limiting its usage to stationary capture in a more confined space. In addition, since the collected images are highly dependent on the naturally occurring light, it will likely collect inconsistent images of the same objects when the lighting conditions are significantly altered. For instance, the spectral responses displayed in a hyperspectral image captured on a sunny day may appear distinct from those captured on a cloudy day.

### 1.2. Related Works

To address the aforementioned limitations, scientists have made several attempts to use active hyperspectral imaging [24,25], comprising two general approaches: (1) using a different imaging mechanism through laser beams, and (2) using external and controlled light sources to illuminate the object of interest. In the first approach, laser beams with various wavelengths can be emitted from the sensors, which would be modulated, and then received by the detector to form a 3D hyperspectral image. These can be designated to operate in a dark environment [26]. However, it has several disadvantages. Firstly, lasers only operate at a limited bandwidth (mainly within the infrared range) and may not cover the full spectrum of the desired bandwidth. Secondly, given the minuscule footprints of these laser beams, it requires very accurate calibration among laser beams having different wavelengths. This incurs a much higher hardware cost for integration and calibration. The second approach adopts coupled light sources that emit full-spectrum light (white light) or time-multiplexed illumination with narrowband lights [27,28]. For example, Park et al., Li et al., and Wang et al. [29,30,31] used mixed RGB light sources to illuminate the scene and a typical RGB camera to capture photos to identify the ideal multiplexing sequence of the spectrum sources. Using a light-diffusing reflector, HyperCam [32] reduces the number of LEDs with extended size. A fixed measure of distance with an enclosed lighting approach aids in avoiding camera-setting biases from one measurement to another. Therefore, for measuring soil and low vegetation, Orlando et al. [33] presented a direct lighting approach with VIS and NIR prototypes. For a variety of applications, an alternative indirect method employing multi-LED structures of various shapes has also been developed. Song et al. [28], for instance, suggested a light pipe with a flip-n-fold method for analyzing the proposed structure of the layout. Several studies considered the use of low-cost LED (light-emitting diode) sources to emit lights at a frequency [34,35,36,37,38,39,40], and the illuminated objects are imaged stationarily at the same frequency to construct bands of data. Some applications that consume hyperspectral data include semantic segmentation with transformers [41], image classifications with graph convolutional networks [42], and hyperspectral-guided stereo matching [43]. Many existing applications often achieve the chromaticity of these lights through a mixture of RGB broadband lights (comprising a wavelength range of 400–700 nm); thus, recovering narrowband responses is an ill-posed problem. However, many of the existing studies directly use such composed lights for downstream applications [35,44,45,46], and in some works, researchers use learning-based methods (convolutional neural networks (CNNs)) [47,48] or Bayesian regularization methods [49] to recover the narrowband spectrums. Moreover, many of these existing works use commercial off-the-shelf RGB cameras [50] as the detector, which is suboptimal, since the RGB filters essentially limit the incoming light and its resulting bands within the visible range.

While these novel approaches and prototype sensors are deemed promising, some are mechanically more difficult to implement, by increasing the cost of the already pricey hyperspectral camera family. This paper presents a potential solution that uses LED coupled with an optical camera, generating economical and compact sensors that can operate in a low-light and confined environment. We offer a prototypically active hyperspectral imaging system that uses synchronized LED lights as active illumination source and demonstrates its viability in typical hyperspectral imaging applications for spectral analysis and material classification.

### 1.3. Contributions

Specifically, this prototype system improves on previous efforts by (1) directly using single-wavelength LEDs on a circular host programmable for illumination, rather than using a mixed RGB to achieve a pseudo-narrowband; (2) utilizing a full spectrum off-the-shelf camera to collect images beyond visible bands; and (3) extensively validating the system through spectral analysis and machine learning-based classification.

In this study, we propose a prototype of an active hyperspectral imaging system that utilizes synchronized LED lights in a low-light/dark environment. The system consists of three modules: (1) an LED-based illumination module; (2) a control module that synchronizes with the shutter of a full-spectrum camera; (3) an image stacking and post-processing module. The system has several advantages in contrast to existing challenges. Firstly, this active sensing system is intended to operate in low-light and dark environments, contradictory to other (passive) systems operating under daylight conditions. Secondly, by changing the illumination settings to only contained sources within the desired wavelengths, the system can easily adjust the spectral range and resolution for data collection and further analysis, reducing the resources required for this process. Thirdly, all of the components used in this system are low-cost, off-the-shelf, and can be potentially manufactured in a compact form to operate in constrained environments, such as in an underground borehole. We evaluate the viability of such a system by collecting hyperspectral images in applications such as rock classification and spectral analysis to distinguish visually similar objects (e.g., printed and natural leaves and spoiled food).

The remaining sections are organized as follows: Section 2 presents our prototype system, which includes subsections of the LED illumination component, camera, and illuminator control, and image gathering; Section 3 explains the experiments, giving the results and validation; Section 4 discusses the anticipated difficulties in its full implementation and the possibility for improvement; Last Section 5 concludes the paper and provides the outlook of the future work.

## 2. Methods and Materials

### 2.1. An Overview of the Proposed System

**Figure 2:**
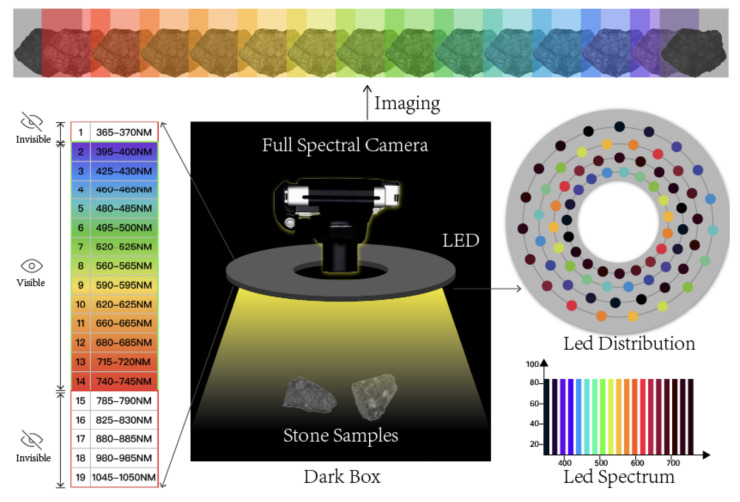
Active hyperspectral imaging system prototype. An overview of the proposed active hyperspectral imaging system (prototype version) collecting images in a low-light/dark environment.

Figure 2 shows an overview of the proposed active hyperspectral system collecting data in a dark environment. It consists of a circular board hosting an array (a total of 76) of single-wavelength (monochromatic) LED light rays and a full-spectrum camera, which are connected through a remote-control module that synchronizes the emitted lights and the camera shutter. The object of interest for imaging is placed under a holding tray in this experimental setup, in which we place different specimens for spectral analysis and machine learning-based material classification (to be introduced in Section 3). The object of interest is kept static throughout the image collection process, conditioned under different LED illumination. In the following few sections, we introduce the design of the LED illuminator, the camera, and the data collection and post-processing components leading to the hyperspectral images.

### 2.2. The LED Illuminator

An LED light is considered a reliable mechanism for illumination [51]. For example, other lighting mechanisms use a combination of tungsten and fluorescent-based illuminators, which inevitably introduce a continuous spectrum or uneven distribution in the spectrum. LED illuminators, in contrast, can provide light spectra of precise and distinctly narrower bandwidths based on the lighting (by exciting specific electrons to photons). Two advantages can be observed due to this phenomenon. Firstly, the luminance of the LED light can be precisely controlled using the amount of current. Secondly, since LED can provide very consistent spectral light, deploying them can be standardized to quantify the spectrum analysis, which would otherwise require calibration. The LED light bulbs are tiny enough to be arranged compactly. Once the prototype (shown in Figure 2) is tested and validated, it can be further compacted for practical usage.

**Figure 3:**
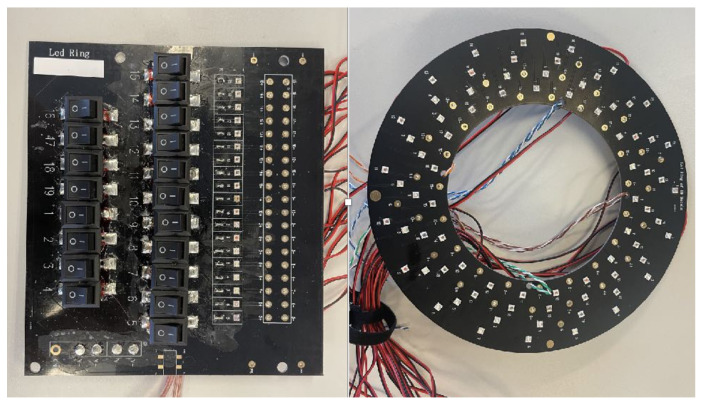
The LED illuminator. Light controller (**left**) and circular LED light panel (**right**).

As shown in Figure 2, the illuminator consists of four concentrically circular rings. Each ring consists of 19 LED lights having the same LED configuration to form a cluster that emits light at a uniform light coverage. The four LED lights in each cluster are placed at 90-degree intervals to create sufficiently strong illumination, at the same time they are distributively positioned to reduce shadow formation by direct lighting. The illuminator can illuminate light rays at 19 unique wavelengths, and its actual implementation was achieved through planting these LED lights onto a circular PCB (printed circuit board), which was powered by a 3W DC supplier and controlled through electrical switches (actual prototype shown in Figure 3). Each LED bulb has a dimension of 3.45 by 3.45 mm, driven by a direct current power supply. The selection of these 19 distinct LEDs aims to cover the widest possible spectrum ranges. To this end, these 19 monochromatic LEDs cover a spectral range of 365 nm–1050 nm, which are expendable depending on the spectral resolution and wavelength of the lights (that can be extended from ultraviolet to 1400 nm in the infrared). Based on the product description and spectrogram [52,53]. The current layout of this prototypical illuminator has a 5nm spectral bandwidth per LED light. These narrowbanded LED lights may potentially generate spectral gaps, and a more desired configuration is to have these LED lights fully cover the spectral range to avoid information loss. In this prototyping stage, we considered current spatial covers broad enough to capture adequate information. As a result, the selected LED lights have their wavelengths as evenly distributed as possible over the spectral range. This is also subject to inventory available at the time of material purchase. As a result, 19 LED lights with unique wavelengths are selected, dividing up the spectral range of 365 nm–1050 nm into approximately equal intervals (as shown in Table 1).

**Figure 4:**
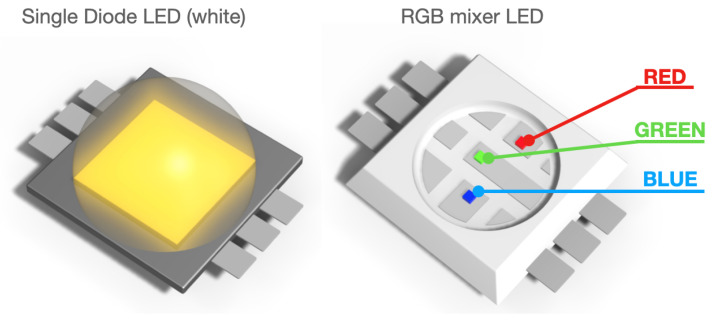
Single diode LED and RGB mixer LED. Illustration of the difference between the single-diode LED and RGB-mixer LED [55].

It should be noted that most previous works use RGB LED mixers, which essentially mix the lighting spectrums of three individually fixed dye diodes that do not cover a spectrum beyond the visible range. In contrast, we directly use a single dye diode that responds to a single electrical current source [54], which can directly emit light at a narrow wavelength bandwidth and a higher spectral purity, operating beyond visible bands (an example of the single diode LED and RGB mixer LED is shown in Figure 4). The HyperCam [32], a single-diode LED source prototype, is similar to the design of our illuminator, but they used a light-diffusive reflector to create uniform illumination from single LED sources. While this reduces the number of LEDs required, there may arise two challenges. Firstly, the strength of illumination may not be sufficient. Secondly, the reflector may distort the spectral purity of the LED lights, adding another anomalous source to the final result. Our design (i.e., utilizing LEDs with uniformly distributed wavelengths) is advantageous in these two aspects.

**Figure 5:**
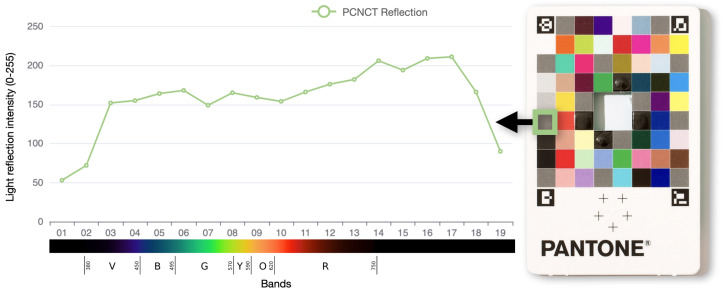
The light intensity of reflection on the PCNCT. The intensity of reflections across different wavelengths on the Pantone Color Match Card (PCNCT). The X-axis refers to the number of bands and wavelengths, the Y-axis represents the reflection intensity of the light from PCNCT (normalized to [0, 255]).

In a dark or low-light environment, the digitized image of objects is determined by the intensity of the LED lighting, the camera settings, and the surface material of the objects. We aim to have consistent absolute light intensity across different wavelengths with the same camera settings. To adjust this, we carried out a test with the Pantone Color Match Card (PCNCT) [56]. For each wavelength of light, we measured the intensity of the reflection from the same color patch (middle gray). If the brightness of various LED lights is similar, the intensity of reflected light should be constant. Figure 5 depicts the measurement of reflective intensity, signifying that the intensity of the reflectance is constant (mainly in the visible bands). Additionally, the reactions in the infrared and ultraviolet ranges are noticeably weaker (a third to a half of the visible intensity). These non-visible portions are difficult to characterize with Pantone cardboard, which results in a diminished reflective intensity.

### 2.3. Camera and Illuminator Control

**Figure 6:**
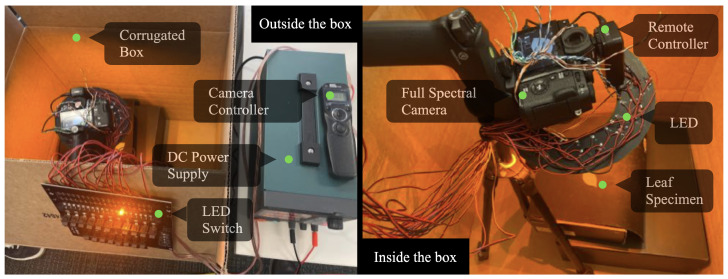
The active hyperspectral experiment setup. The active hyperspectral imaging experiment is carried out in a dark environment. Different components of this experiment setup are marked in the figure.

A normal RGB camera using an infrared mirror filter may significantly downgrade the received spectral quality while truncating the spectral information beyond the visible range. Therefore, to fully capture the reflected light via our active illuminator, we deploy a full-spectrum camera capable of receiving spectrums at a wider range than the normal RGB camera. In the meantime, as verified in Figure 5 (Section 2.2), considering the intensity of the emitted lights is mostly consistent, we would be able to collect hyperspectral images without any need for calibration. Specifically, for our proposed prototypical system, we used the Fujifilm X-T1 IR model, equipped with a CMOS sensor but excluding an infrared cut-off filter. As a result, this camera can capture light from the ultraviolet (UV), visible, and infrared (IR) portions of the spectrum (approximately 380 nm–1000 nm), and can provide approximately twice the amount of spectral information by an RGB color camera. We avoided using any lens filter attached to the camera to allow the reception of any or all incoming light, which is meticulously controlled by our illuminator. A remote control is linked to the power switch and shutter as shown in Figure 6 (Section 3). The camera parameters are preset to accommodate low-lit and dark environments. The exposure time was set to 1/8th second, and the aperture was set to f/14.0 to achieve a trade-off between the amount of light received and the depth of the field. A moderate ISO sensitivity of 800 was chosen to reduce noise and increase brightness. A fixed focal length of 90 mm was used to capture close-range objects. These parameters can be adjusted to accommodate different lighting conditions.

### 2.4. Image Collection

While capturing a hyperspectral image of an object, the system will loop over all 19 LED light channels and capture a panchromatic image of each. Thus, the capturing process of a hyperspectral image of the object will go through the following steps. The PCB controls an LED, which is connected to the direct current (DC) power supply. It has programmed an automatic logic to run the subsequent LED circuit whenever the power is turned on and off. We use the DC power supply’s software to create an automated test sequence to switch LEDs after a certain amount of time. The sequence also controls the camera by using the FUJIFILM Camera Remote app, which is an application provided by FUJIFILM. This application can operate wireless digital cameras to shoot images with synchronized LED lights. Theoretically, the capturing interval can be decreased to milliseconds, which shall require high-speed synchronization between the illuminator and camera.

## 3. Experiment

Two sets of experiments were performed to validate the prototype of the proposed camera. The first set of experiments aimed to determine if our proposed prototype could provide spectral information beyond the capturing properties of typical RGB images. This was done by qualitative analysis of the objects’ spectral responses that were easily distinguished by hyperspectral cameras. This included analyzing the visual augmentation of hyperspectral imaging for identifying fresh and wilted strawberries (Experiment-I) and real and printed (picture) leaves (Experiment-II). The second set of experiments examined the sufficiency of the resulting hyperspectral image characterizations of objects and facilitation of machine-learning applications recognizing objects that were complex to differentiate by merely using their RGB images. In this experiment, we collected several visually similar rock specimens, imaged them through our system, and performed a machine learning-based classification to identify different types of rocks (Experiment-III). All of these experiments were performed using the proposed prototype setup as shown in Figure 6.

### 3.1. Experiment I—Identifying Fresh and Wilted Strawberry

**Figure 7:**
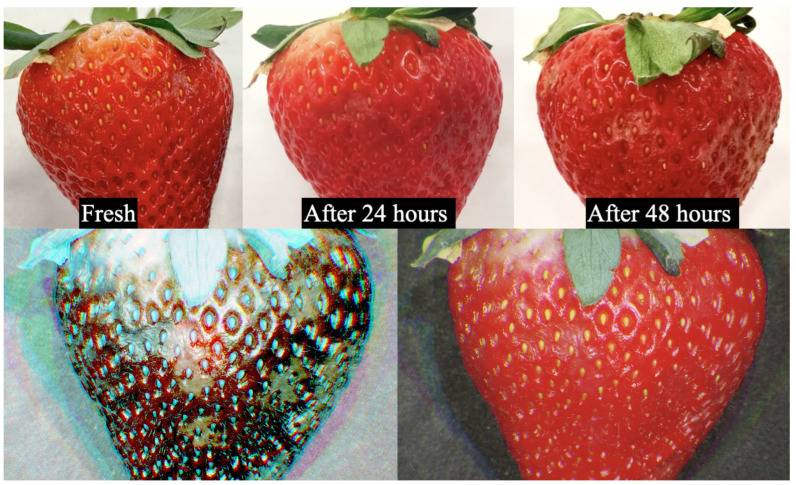
The same strawberry was taken by an RGB camera and active hyperspectral camera. The first row shows pictorial comparisons of the conditions in terms of fresh (**left**), post-24 h (**middle**), and 48 h (**right**) of the same strawberry specimen from a supermarket captured by an RGB camera. The second row shows a pseudo-colored image on the left, generated from the most distinctive bands (1, 3, and 4) of the same strawberry captured by our active low-cost hyperspectral camera. The same strawberry has generated a pseudo-colored image (on the right) from the most representative RGB bands (4, 7, and 11), captured by our active low-cost hyperspectral camera.

The hyperspectral camera has been used as an effective tool in the food industry to detect the level of freshness and identify potential contamination of food products as a measure to prevent complaints and recalls [57]. In this experiment, we examined the freshness of a strawberry through our proposed camera. We kept a fresh strawberry in a room environment and regularly captured images using our camera system. Specifically, we sampled the image at 0 h (fresh), 24 h, and 48 h, using an RGB camera and the proposed hyperspectral camera. All three RGB images had the same camera settings, specifically an ISO of 320, an aperture of 2.2f, and an exposure time of 1/8 s. Our active hyperspectral imaging collected 3 images with a total of 19 bands. The comparative results are shown in Figure 7. It can be seen that the RGB images did not show much difference in chromaticity, although part of the strawberry shows somewhat textural differences. In contrast, the hyperspectral images (visualized using selected bands) show distinctive spectral differences. It clearly shows the benefits of hyperspectral imaging to facilitate easy detecting algorithms for identifying wilted regions of the fruits. Furthermore, it verifies that our proposed active hyperspectral imaging collects expected images. The selection criteria of the bands for visualization in Figure 7 were based on the principle that (while representing RGB) different bands from our active hyperspectral system would highlight the overripe parts. We selected wavelengths lower than 450 nm, which corresponded to the changes in pigmentation, chlorophyll, and moisture content of fresh and 24 h strawberries [58,59], to demonstrate the overripe parts. Bands 4, 7, and 11—the most representative bands—were selected to recreate the strawberry in RGB [60].

**Figure 8:**
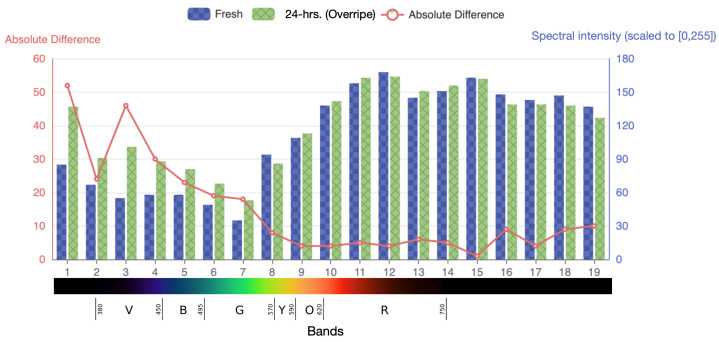
The light intensities in different bands of fresh and overripe strawberries. The light intensities in different bands of fresh (blue bar) and overripe (green bar) strawberries are demonstrated. The red line represents the absolute difference between the two light-intensity values. The Y-axis represents the light reflective intensity of the strawberry. The X-axis displays the number of bands, with each band’s corresponding spectrum represented in RGB color for ease of reference.

We further evaluate the differences in the spectrum by comparing fresh and post-24 h conditions of the strawberry. We extract the mean spectral responses of the object for these 19 bands, as shown in Figure 8. These responses are comparable since the luminance of the light is consistent in each captured image. As seen in Figure 8, the absolute differences between the spectral responses suggest that bands 1, 3, and 4, are the most distinctive and consistent with our earlier analysis, highlighting the overripe part of the strawberry.

### 3.2. Experiment II—Leaf Experiment

**Figure 9:**
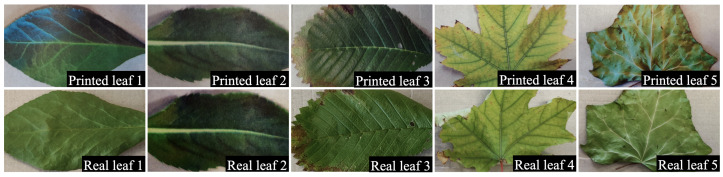
Comparison of printed and real leaf by RGB camera. Part of the comparison between printed and real leaf pairs was taken from an RGB camera.

Experiment II aims to verify the spectrum competence of hyperspectral imaging over typical broadband RGB images. Images of printed and real leaves are used in this experiment using a typical RGB camera and the proposed hyperspectral camera. We selected six diverse leaves in terms of shape, texture, and appearance. The same leaves were scanned and printed on A4 papers. Since these leaves underwent scanning and printing processes, their spectral properties are expected to be notably different from their printed counterparts. Images were taken following the same camera settings (i.e., ISO, aperture, and exposure time) as used in Experiment-I. Figure 9 shows the RGB images of these leaves and their printed counterparts, which show no apparent visual differences between the real and printed leaves.

**Figure 10:**
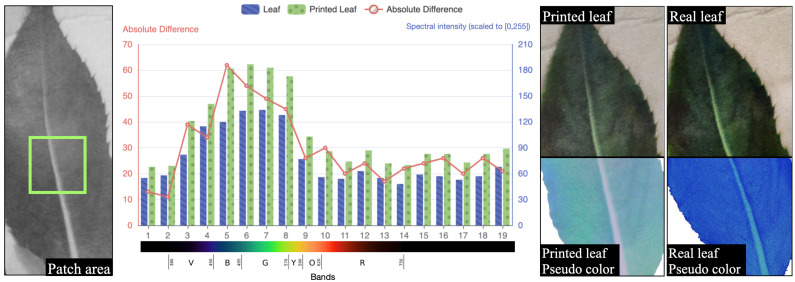
The spectral analysis between the printed and real leaf from the same patch area. The combined image shows the spectral analysis between the printed and the real leaf (image collage on the right) from the same patch area (leftmost image). The chart highlighting spectral analysis (image at the center) is the same as Figure 8. The comparisons between the printed (second to the extreme right image) and real (rightmost image) leaves are made with RGB images, and the most distinct bands (5, 6, and 7) of the pseudo-colored image.

We further analyze the spectral differences between these printed and real leaves using the images captured by our proposed hyperspectral camera. As depicted in Figure 10, the spectral response of one of the leaf pairs is analyzed, which shows that a few bands are distinctively different between the printed and the real leaf. For example, the paper constituents in the printed leaf generate a notable spectral reflectance peak of around 500 nm, deviating from the spectral response of a real leaf [61].

**Figure 11:**
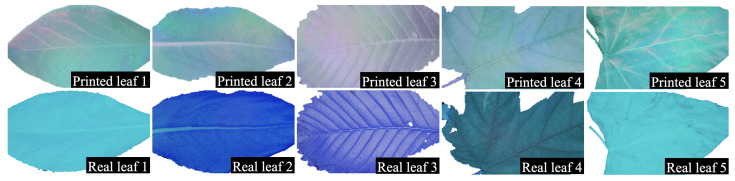
Comparison of printed and real leaf by the hyperspectral camera. Synthetic RGB images of leaf pairs generated from the bands have the most significant absolute differences between the real and printed leaf images as collected by our active and low-cost hyperspectral camera.

We visualize the image using bands 5, 6, and 7 (the corresponding wavelengths are shown in Table 1) for the pairs of leaves, as shown in Figure 11. It is clearly shown that these bands can be used to sufficiently differentiate printed and real leaves. The synthetic RGB image for printed leaves has a diverse gradient of chromatic light reflection (refer to Figure 11) due to the injection of fluorescent ink on the paper [61]. On the other hand, different leaves have reflections of diverse light intensity due to the varying proportions of chlorophyll [62].

### 3.3. Experiment III—Stone Specimen Experiment

**Figure 12:**
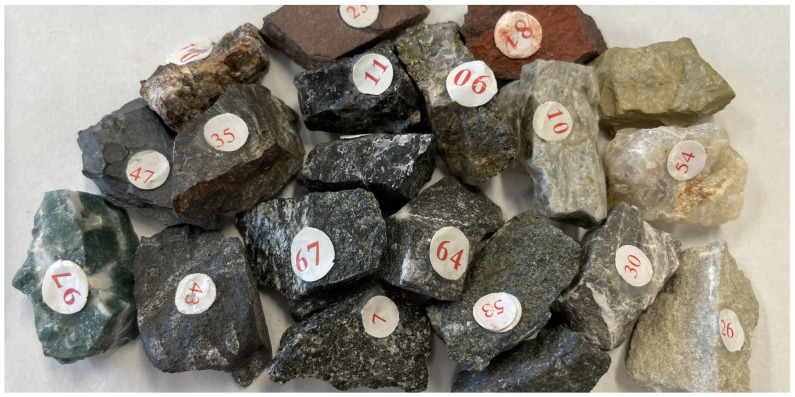
Stone samples. Samples of stones showing inter-class similarity in terms of their visual appearance.

The hyperspectral images can provide more spectral information about machine learning and image analysis. In this experiment, hyperspectral imaging has been used to address the challenges in stone identification using machine learning approaches. Specifically, 20 stones of different categories were collected as samples. Some stones were visually highly similar (as shown in Figure 12). These stones include basalt, obsidian, perlite, plagiogranite, shale, aleuritic-textured shale, arenite, limestone, siliceous rock, carbonaceous limestone, slate, quartzite, anhydrite, serpentine, graphite, alunite, hematite, chalcopyrite, and agate. Images were recorded following consistent camera settings (i.e., ISO, aperture, and exposure time) as used in Experiment-I.

The images of these twenty stones were captured using an RGB and our proposed hyperspectral camera, on three sides, each face recorded to create the dataset. It results in a total of 20 (number of classes) × 3 (different sides) × 19 (bands) hyperspectral images, as well as the corresponding RGB images. By cropping stone patches from these images at 100 × 100 pixels, 114,000 samples have been generated, of which 80% are used for training and 20% for testing.

To resolve this experiment of the twenty-sample classification, we train a random forest with n estimators/trees (*n* = 10). The 19-band input imagery patch was summarized to the average/median/Gaussian weighted mean value, and the stone’s category is the resulting output. The result shown in Table 2 indicates, the model trained with hyperspectral images has about 90% accuracy rate, which is significantly higher than the model trained with only RGB images, having approximately 70% accuracy rate.

## 4. Discussion

During the design and experiment phase, we verified that this prototype could function as a typical hyperspectral camera in acquiring further spectral information, leading to better classification results on objects that typically pose complexities when using only RGB imageries. Specifically, these experiments were performed in a dark and constrained environment. This prototype only demonstrates the feasibility of such a system. Although some challenges exist, there are huge scopes for improvement to develop this system for practical usage, i.e., to be more efficient and portable (compact) for data collection in a confined space.

A practical system will require faster image acquisition to avoid motion blurring and more collections could be yielded at a specific period. Data collection time differs significantly between our designed prototype and major passive hyperspectral systems. Minimizing the capture time among bands requires significant hardware design for perfect synchronization between the shutter and the programmable lighting. The current prototype system did not optimize this component, due to which it required about 20 s to collect the entire image collection. For example, if moderately optimized, imaging one spectrum may take only 1/8th of a second (i.e., a single shot). Thus, the time required to collect all 19 bands can be reduced to 2.375 s. Additionally, our prototype requires an image-stacking process, which could be easily removed once the prototype is further developed by using a more automated synchronization and data-storing module. Moreover, the quality of the data can be improved by adopting various means. The significant measure is to increase the number of spectrum bands. This can, however, become challenging since our data acquisition system linearly captures bands using LEDs. More LEDs lead to higher logistical and space costs and amplified collection time. We believe this can be moderately addressed by splitting the spectrum range into two or more sets of an active lighting system, which would not further increase the lighting density, or by using LEDs with variable wavelengths to facilitate more compact illumination systems. Furthermore, if both efficiency and compactness are addressed, the system would facilitate a better scenario, for example, enabling the camera to probe dark and confined spaces and stream live data for machine learning applications in real time and onsite decision-making. In our future efforts, we expect to address these aspects through more advanced manufacturing, system integration, and data analytics.

## 5. Conclusions

Hyperspectral cameras are great tools for object identification, yet most are passive imaging systems that are unable to work in darker environments. This paper demonstrates our proposed prototype of the active hyperspectral system, which can be used in a dark environment. In contrast to pre-existing solutions, our proposed low-cost system uses accurate narrow band illuminators across a dense spectrum range (that offers 19 bands with maximum coverage extension) mounted on a specifically designed ring pattern, coupled with a synchronizer and a full-spectrum camera. We assessed the system potentiality of this proposed active hyperspectral camera comprehensively through three experiments (1) freshness detection of food, (2) comparison between real and printed leaves, and (3) identification and categorization of rock specimens (refer to Section 3).

The results of the experiments suggested the following conclusions: First, accurate spectral analysis is achievable with low-cost LED lights. It gives the chance to develop low-cost, lightweight systems to be able to collect objects with better mobility. Second, our prototype hyperspectral system has the ability to discern different objects that are not succeeded by standard RGB cameras. Specifically, we observed that the change in the freshness of strawberries is readily detectable at a time resolution of 24 h or less.

Real and printed leaves show distinctive spectral signatures under our camera systems while denoting visual similarity under a standard RGB camera. Coupled with simple machine learning approaches, the images obtained from our camera system achieves higher classification accuracy (+22% max) as compared to images obtained via a typical RGB camera. It has been demonstrated that such a system is feasible for low-light conditions. Nevertheless, during our experiments, we also observed several challenges, including the shadow effects of images projected from different bands within close ranges, a time delay of capture among bands, as well as the challenges of manufacturing such a system into a compact form to facilitate its usage in confined spaces, for example in boreholes. Therefore, in our future work studies, we will aim to enhance the compactness and integration of the system, which will yield a higher readiness level.

## 6. Disclaimer

Mentioning the brands does not constitute an endorsement from the authors.

## Figures and Tables

**Table 1:** Spectral band number with its corresponding wavelength of the LED lights.

Band(#)	1	2	3	4	5	6	7
**Wavelength (nm)**	365–370	395–400	425–430	460–465	480–485	495–500	520–525
**Band(#)**	**8**	**9**	**10**	**11**	**12**	**13**	**14**
**Wavelength (nm)**	560–565	590–595	620–625	660–665	680–685	715–720	740–745
**Band(#)**	**15**	**16**	**17**		**18**		**19**
**Wavelength (nm)**	785–790	825–830	880–885		980–985		1045–1050

**Table 2:** The performance comparison of RF (random forest classifier) results.

Data	Mean Value	Median Value	Gaussian Weighted Mean Value
RGB image	74.4%	75.1%	68.8%
Hyperspectral image	90.2%	91.6%	91.0%
Difference	+15.8%	+16.5%	+22.2%

## Data Availability

The data presented in this study are openly available in Datadryad at https://doi.org/10.5061/dryad.n02v6wx17, accessed on 26 December 2022.

## Abbreviations

The following abbreviations are used in this manuscript:

LED	Light-Emitting Diode
CNN	Convolutional Neural Networks
PCB	Printed Circuit Board
DC	Direct Current
PCNCT	Pantone Color Match Card
CMOS	Complementary Metal Oxide Semiconductor
UV	Ultraviolet
IR	Infrared
HDR	High Dynamic Range

## References

[1] Sowmya V., Soman K.P., Hassaballah M., Hassaballah M., Hosny K.M. (2019). Hyperspectral Image: Fundamentals and Advances. Recent Advances in Computer Vision: Theories and Applications.

[2] Li J., Pei Y., Zhao S., Xiao R., Sang X., Zhang C. (2020). A Review of Remote Sensing for Environmental Monitoring in China. Remote Sens..

[3] Song W., Song W., Gu H., Li F. (2020). Progress in the Remote Sensing Monitoring of the Ecological Environment in Mining Areas. Int. J. Environ. Res. Public Health.

[4] Liaghat S., Balasundram S. (2010). A Review: The Role of Remote Sensing in Precision Agriculture. Am. J. Agric. Biol. Sci..

[5] Ambrose A., Kandpal L.M., Kim M.S., Lee W.H., Cho B.K. (2016). High Speed Measurement of Corn Seed Viability Using Hyperspectral Imaging. Infrared Phys. Technol..

[6] Mehta N., Sahu S.P., Shaik S., Devireddy R., Gartia M.R. (2021). Dark-Field Hyperspectral Imaging for Label Free Detection of Nano-Bio-Materials. WIREs Nanomed. Nanobiotechnol..

[7] Raza A., Dumortier D., Jost-Boissard S., Cauwerts C., Dubail M. (2023). Accuracy of Hyperspectral Imaging Systems for Color and Lighting Research. LEUKOS.

[8] Roggo Y., Edmond A., Chalus P., Ulmschneider M. (2005). Infrared Hyperspectral Imaging for Qualitative Analysis of Pharmaceutical Solid Forms. Anal. Chim. Acta.

[9] Lassalle G., Credoz A., Hédacq R., Fabre S., Dubucq D., Elger A. (2018). Assessing Soil Contamination Due to Oil and Gas Production Using Vegetation Hyperspectral Reflectance. Environ. Sci. Technol..

[10] Saeedifar M., Mansvelder J., Mohammadi R., Zarouchas D. (2019). Using Passive and Active Acoustic Methods for Impact Damage Assessment of Composite Structures. Compos. Struct..

[11] Dong L., Pei Z., Xie X., Zhang Y., Yan X. (2022). Early Identification of Abnormal Regions in Rock-Mass Using Traveltime Tomography. Engineering.

[12] Zhang Y.B., Yao X.L., Liang P., Wang K.X., Sun L., Tian B.Z., Liu X.X., Wang S.Y. (2021). Fracture Evolution and Localization Effect of Damage in Rock Based on Wave Velocity Imaging Technology. J. Cent. South Univ..

[13] Lacar F.M., Lewis M., Grierson I. Use of Hyperspectral Imagery for Mapping Grape Varieties in the Barossa Valley, South Australia. Proceedings of the IGARSS 2001, Scanning the Present and Resolving the Future, IEEE 2001 International Geoscience and Remote Sensing Symposium (Cat. No.01CH37217).

[14] Asner G.P., Knapp D.E., Kennedy-Bowdoin T., Jones M.O., Martin R.E., Boardman J.W., Field C.B. (2007). Carnegie Airborne Observatory: In-Flight Fusion of Hyperspectral Imaging and Waveform Light Detection and Ranging for Three-Dimensional Studies of Ecosystems. J. Appl. Remote Sens..

[15] Ferwerda J.G. (2005). Charting the Quality of Forage: Measuring and Mapping the Variation of Chemical Components in Foliage with Hyperspectral Remote Sensing.

[16] Mars J.C., Crowley J.K. (2003). Mapping Mine Wastes and Analyzing Areas Affected by Selenium-Rich Water Runoff in Southeast Idaho Using AVIRIS Imagery and Digital Elevation Data. Remote Sens. Environ..

[17] Zhang M., He T., Li G., Xiao W., Song H., Lu D., Wu C. (2021). Continuous Detection of Surface-Mining Footprint in Copper Mine Using Google Earth Engine. Remote Sens..

[18] Yu L., Xu Y., Xue Y., Li X., Cheng Y., Liu X., Porwal A., Holden E.J., Yang J., Gong P. (2018). Monitoring Surface Mining Belts Using Multiple Remote Sensing Datasets: A Global Perspective. Ore Geol. Rev..

[19] Themelis G., Yoo J.S., Ntziachristos V. (2008). Multispectral Imaging Using Multiple-Bandpass Filters. Opt. Lett..

[20] Du H., Tong X., Cao X., Lin S. A Prism-Based System for Multispectral Video Acquisition. Proceedings of the 2009 IEEE 12th International Conference on Computer Vision.

[21] Gómez-Sanchis J., Lorente D., Soria-Olivas E., Aleixos N., Cubero S., Blasco J. (2014). Development of a Hyperspectral Computer Vision System Based on Two Liquid Crystal Tuneable Filters for Fruit Inspection. Application to Detect Citrus Fruits Decay. Food Bioprocess Technol..

[22] Renhorn I.G.E., Bergström D., Hedborg J., Letalick D., Möller S. (2016). High Spatial Resolution Hyperspectral Camera Based on a Linear Variable Filter. Opt. Eng..

[23] Functionality of Measuring Systems—LLA Instruments GmbH & Co KG. https://www.lla-instruments.de/en/how-it-works-en/functionality-of-measuring-systems.html.

[24] Fischer C., Kakoulli I. (2006). Multispectral and Hyperspectral Imaging Technologies in Conservation: Current Research and Potential Applications. Stud. Conserv..

[25] Boldrini B., Kessler W., Rebner K., Kessler R.W. (2012). Hyperspectral Imaging: A Review of Best Practice, Performance and Pitfalls for in-Line and on-Line Applications. J. Near Infrared Spectrosc..

[26] Guo Z., Liu Y., Zheng X., Yin K. (2019). Active Hyperspectral Imaging with a Supercontinuum Laser Source in the Dark. Chin. Phys. B.

[27] Multispectral Imaging Systems. https://spectraldevices.com/collections/multispectral-imaging-system.

[28] Song J.Y., Bian L.F., Sun X.M., Ding Z., Yang C. (2022). Design of Active Hyperspectral Light Source Based on Compact Light Pipe with LED Deflection Layout. Opt. Laser Technol..

[29] Park J.I., Lee M.H., Grossberg M.D., Nayar S.K. Multispectral Imaging Using Multiplexed Illumination. Proceedings of the 2007 IEEE 11th International Conference on Computer Vision.

[30] Li H.N., Feng J., Yang W.P., Wang L., Xu H.B., Cao P.F., Duan J.J. Multi-Spectral Imaging Using LED Illuminations. Proceedings of the 2012 5th International Congress on Image and Signal Processing.

[31] Wang H., Hu Y., Ma X., Sun J., Sun X., Chen D., Zheng X., Li Q. (2019). An Active Hyperspectral Imaging System Based on a Multi-LED Light Source. Rev. Sci. Instrum..

[32] Goel M., Whitmire E., Mariakakis A., Saponas T.S., Joshi N., Morris D., Guenter B., Gavriliu M., Borriello G., Patel S.N. (2015). HyperCam: Hyperspectral Imaging for Ubiquitous Computing Applications. Proceedings of the 2015 ACM International Joint Conference on Pervasive and Ubiquitous Computing (UbiComp’15).

[33] Orlando S., Minacapilli M., Sarno M., Carrubba A., Motisi A. (2022). A Low-Cost Multispectral Imaging System for the Characterisation of Soil and Small Vegetation Properties Using Visible and near-Infrared Reflectance. Comput. Electron. Agric..

[34] Tschannerl J., Ren J., Zhao H., Kao F.J., Marshall S., Yuen P. (2019). Hyperspectral Image Reconstruction Using Multi-colour and Time-multiplexed LED Illumination. Opt. Lasers Eng..

[35] Mo C., Kim G., Lee K., Kim M.S., Cho B.K., Lim J., Kang S. (2014). Non-Destructive Quality Evaluation of Pepper (*Capsicum annuum* L.) Seeds Using LED-induced Hyperspectral Reflectance Imaging. Sensors.

[36] JSSS (2022). Near-infrared LED System to Recognize Road Surface Conditions for Autonomous Vehicles. https://jsss.copernicus.org/articles/11/187/2022/.

[37] Casselgren J., Rosendahl S., Sjödahl M., Jonsson P. (2016). Road Condition Analysis Using NIR Illumination and Compensating for Surrounding Light. Opt. Lasers Eng..

[38] Thörnberg B. The Material Imaging Analyzer MIA. Proceedings of the 2022 IEEE Sensors Applications Symposium (SAS).

[39] Xu J.L., Sun D.W. (2017). Identification of Freezer Burn on Frozen Salmon Surface Using Hyperspectral Imaging and Computer Vision Combined with Machine Learning Algorithm. Int. J. Refrig..

[40] Xu J.L., Riccioli C., Sun D.W. (2017). Comparison of Hyperspectral Imaging and Computer Vision for Automatic Differentiation of Organically and Conventionally Farmed Salmon. J. Food Eng..

[41] Hong D., Han Z., Yao J., Gao L., Zhang B., Plaza A., Chanussot J. (2022). SpectralFormer: Rethinking Hyperspectral Image Classification With Transformers. IEEE Trans. Geosci. Remote Sens..

[42] Hong D., Gao L., Yao J., Zhang B., Plaza A., Chanussot J. (2021). Graph Convolutional Networks for Hyperspectral Image Classification. IEEE Trans. Geosci. Remote Sens..

[43] Chang J.R., Chen Y.S. Pyramid Stereo Matching Network. Proceedings of the 2018 IEEE/CVF Conference on Computer Vision and Pattern Recognition.

[44] Lohumi S., Lee H., Kim M.S., Qin J., Kandpal L.M., Bae H., Rahman A., Cho B.K. (2018). Calibration and Testing of a Raman Hyperspectral Imaging System to Reveal Powdered Food Adulteration. PLoS ONE.

[45] Abdel-Rahman F., Okeremgbo B., Alhamadah F., Anthony K., Saleh M. (2017). Caenorhabditis Elegans as a Model to Study the Impact of Exposure to Light Emitting Diode (LED) Domestic Lighting. J. Environ. Sci. Health Part A Toxic/Hazardous Subst. Environ. Eng..

[46] Li S.X. (2018). Filter Selection for Optimizing the Spectral Sensitivity of Broadband Multispectral Cameras Based on Maximum Linear Independence. Sensors.

[47] Yu C., Han R., Song M., Liu C., Chang C.I. (2020). A Simplified 2D-3D CNN Architecture for Hyperspectral Image Classification Based on Spatial–Spectral Fusion. IEEE J. Sel. Top. Appl. Earth Obs. Remote Sens..

[48] Yang X., Ye Y., Li X., Lau R.Y.K., Zhang X., Huang X. (2018). Hyperspectral Image Classification With Deep Learning Models. IEEE Trans. Geosci. Remote Sens..

[49] Zhang Y., Duijster A., Scheunders P. (2012). A Bayesian Restoration Approach for Hyperspectral Images. IEEE Trans. Geosci. Remote Sens..

[50] Oh S.W., Brown M.S., Pollefeys M., Kim S.J. Do It Yourself Hyperspectral Imaging with Everyday Digital Cameras. Proceedings of the 2016 IEEE Conference on Computer Vision and Pattern Recognition (CVPR).

[51] Khan M.N. (2020). Understanding Led Illumination.

[52] Deep Red LED, 3535 Led Chip, Hyper Red Led. https://www.moon-leds.com/product-3535-deep-red-660nm-smd-led.html.

[53] Royal Blue 3535 SMD LED, 3535 LED, Blue Led. https://www.moon-leds.com/product-royal-blue-450nm-3535-smd-led.html.

[54] Huang Y., Cohen T.A., Luscombe C.K. (2021). Naturally Derived Organic Dyes for LED Lightings of High Color Rendering and Fidelity Index. ChemRxiv.

[55] LED Correlated Color Temperature and 5050 LEDs. https://www.boogeylights.com/understanding-led-color-temperature/.

[56] PANTONE® USA|Pantone Color Match Card (PCNCT). https://www.pantone.com/pantone-color-match-card.

[57] Bhargava A., Bansal A. (2021). Fruits and Vegetables Quality Evaluation Using Computer Vision: A Review. J. King Saud Univ. Comput. Inf. Sci..

[58] Nagata M., Tallada J.G., Kobayashi T. (2006). Bruise Detection Using NIR Hyperspectral Imaging for Strawberry (*Fragaria × ananassa* Duch.). Environ. Control Biol..

[59] Cheng J.H., Sun D.W., Nagata M., Tallada J.G., Sun D.W. (2016). Chapter 13—Quality Evaluation of Strawberry. Computer Vision Technology for Food Quality Evaluation.

[60] Mihai D., Strǎjescu E. (2007). From Wavelength to R G B Filter. UPB Sci. Bull..

[61] Krauz L., Páta P., Kaiser J. (2022). Assessing the Spectral Characteristics of Dye- and Pigment-Based Inkjet Prints by VNIR Hyperspectral Imaging. Sensors.

[62] Borsuk A.M., Brodersen C.R. (2019). The Spatial Distribution of Chlorophyll in Leaves. Plant Physiol..

